# Comparative proteome analysis of psychrophilic *versus *mesophilic bacterial species: Insights into the molecular basis of cold adaptation of proteins

**DOI:** 10.1186/1471-2164-10-11

**Published:** 2009-01-08

**Authors:** Raghu Prasad Rao Metpally, Boojala Vijay B Reddy

**Affiliations:** 1 Laboratory of Bioinformatics and in Silico Drug Design, Computer Science Department, Queens College of City University of New York, 65-30 Kissena Blvd., Flushing, NY 11367, USA

## Abstract

**Background:**

Cold adapted or psychrophilic organisms grow at low temperatures, where most of other organisms cannot grow. This adaptation requires a vast array of sequence, structural and physiological adjustments. To understand the molecular basis of cold adaptation of proteins, we analyzed proteomes of psychrophilic and mesophilic bacterial species and compared the differences in amino acid composition and substitution patterns to investigate their likely association with growth temperatures.

**Results:**

In psychrophilic bacteria, serine, aspartic acid, threonine and alanine are overrepresented in the coil regions of secondary structures, whilst glutamic acid and leucine are underrepresented in the helical regions. Compared to mesophiles, psychrophiles comprise a significantly higher proportion of amino acids that contribute to higher protein flexibility in the coil regions of proteins, such as those with tiny/small or neutral side chains. Amino acids with aliphatic, basic, aromatic and hydrophilic side chains are underrepresented in the helical regions of proteins of psychrophiles. The patterns of amino acid substitutions between the orthologous proteins of psychrophiles *versus *mesophiles are significantly different for several amino acids when compared to their substitutions in orthologous proteins of within the mesophiles or psychrophiles.

**Conclusion:**

Current results provide quantitative substitution preferences (or avoidance) of amino acids that lead to the adaptation of proteins to cold temperatures. These finding would help future efforts in selecting mutations for rational design of proteins with enhanced psychrophilic properties.

## Background

Microorganisms that live under forbidding conditions are called extremophiles, whose discovery points out the unique adaptability of primitive life-forms. These microorganisms are grouped according to their optimal growth conditions in which they exist such as acidophiles (exhibiting optimum growth in acidic pH conditions), alkaliphiles (thriving in alkaline pH conditions), barophiles (surviving under great pressures), endoliths (living in deep inside rocks), halophiles (thriving in high salt concentrations), psychrophiles (optimal temperature below 20°C), and the thermophiles (optimal temperature between 45–80°C), hyperthermophiles (optimal temperature above 80°C) [1]. The largest coverage of known extremophile conditions of the earth's biosphere is below 10°C. For example, three fourths of earth is covered by oceans, which maintain an average temperature of one to three degrees centigrade. Furthermore, the vast land areas of the Arctic and Antarctic are permanently frozen throughout the year [1]. Other few examples of cryo habitats include cold deserts, high alpine soils, sea ice, cold caves, marine sediments, permafrost soils, glacier, snow etc.

The majority of known psychrophiles belong to varieties of archaea and bacteria, and a few species of yeast, fungi and algae [2]. The ability to thrive at life-endangering effects of low temperatures, close to freezing point of water, requires a vast array of adaptations from all their cellular components, including their membranes, energy-generating systems, protein synthesis machinery, biodegradative enzymes and the components responsible for nutrient uptake etc., to maintain metabolism, sustain growth and reproduction compatible with life in these low temperature conditions [3,4]. Having evolved with special mechanisms, the psychrophiles successfully colonized these niches [2,5]. Psychrophilic proteins display sequences and structures comparable with those of their meso and (hyper) thermophilic homolog's, especially enzymes with their ability to work efficiently as catalysts at low temperatures [6]. The thermolability of these proteins at moderate temperatures warrant tremendous industrial applications in biotechnology, bioremediation, food, textiles, detergents bio-catalysis under low-water conditions and detergents etc [5-9].

Due to above facts, historically starting from mid-1970's, much attention was paid mainly to sequence and structural attributes contributing to adaptation of proteins (mainly enzymes) to high temperature conditions. Many investigators have compared sequence and structure-based parameters among thermophilic and mesophilic proteins [10]. With the advent of pioneering efforts in late 1990's in solving three dimensional structures of cryophilic enzymes such as alpha-amylase [11]; alkaline protease [12]; triose phosphate isomerase [13]; malate dehydrogenase [14] from Antarctic microorganisms, and due to handful of available structures in the protein data bank (PDB), groups have focused to address the structural basis of proteins in cold adaptation [4,15-20].

The steady increase in sequencing of proteomes of extremophiles has opened many new avenues in understanding adaptations to extreme conditions [16,21-25]. A comprehensive comparison of global amino acid preferences and substitution patterns as deduced from proteomes of different organisms is now possible [26-28]. Using homologous sequences, clustering along with various statistical methods; we conducted an extensive analysis of proteomes of psychrophilic, mesophilic, thermophilic and hyperthermophilic microorganisms to examine a possible correlation of amino acid substitution patterns with adaptation to their respective optimal growth conditions. In this manuscript we discuss the results from comparative analysis of fully sequenced proteomes of six members from each of psychrophilic and mesophilic organisms.

## Results

On average we analyzed 2,816 proteins with 875,219 amino acids per proteome of mesophiles and 3665 proteins with 1,169,678 amino acids per proteome of psychrophiles. The amino acid (AA) frequencies given in Table 1 show that some of the AA differed significantly in psychrophile proteomes when compared to mesophile proteomes. When compared to psychrophiles, the mesophile proteomes show larger standard deviation for residues indicating that the six proteomes of mesophiles we used are considerably more divergent than the proteomes of psychrophiles. The frequencies of individual amino acids as well as property groups were further analyzed with student *t-test*.

**Table 1:** The composition of individual amino acids and property groups in protein sequences of psychrophilic and mesophilic proteomes.

**Amino Acids**	**Psychrophiles^a^**								**Mesophiles^b^**								
	**P1**	**P2**	**P3**	**P4**	**P5**	**P6**	**Avg**	**SD**	**M1**	**M2**	**M3**	**M4**	**M5**	**M6**	**Avg**	**SD**	**t-test**
**Ala (A)**	8.1	8.5	9.2	8.4	8.9	12.3	9.2	1.6	8.3	6.8	9.5	8.4	9.1	6.7	8.1	1.2	**1.373**
**Cys (C)**	1.0	1.4	0.9	1.1	1.0	0.7	1.0	0.2	1.0	1.1	1.2	1.0	1.1	0.6	1.0	0.2	0.146
**Asp (D)**	5.6	5.1	6.0	5.4	5.8	5.3	5.5	0.3	5.0	4.8	5.1	5.0	5.0	5.9	5.1	0.4	**1.801**
**Glu (E)**	5.9	6.3	5.5	5.9	5.8	5.6	5.8	0.3	6.5	6.9	5.8	6.1	6.2	6.6	6.3	0.4	**-2.547**
**Phe (F)**	4.4	4.3	3.7	4.4	4.3	3.4	4.1	0.4	4.4	5.4	3.9	4.0	4.1	4.3	4.4	0.6	-1.006
**Gly (G)**	6.4	7.5	6.6	6.5	6.8	8.4	7.1	0.8	6.7	5.8	7.4	7.3	6.7	6.3	6.7	0.6	0.922
**His (H)**	2.2	2.0	2.3	2.1	2.3	1.9	2.1	0.2	2.1	2.1	2.3	1.9	2.4	1.8	2.1	0.2	0.206
**Ile (I)**	7.2	7.1	6.9	7.4	6.2	5.0	6.6	0.9	7.1	7.2	6.0	6.3	6.0	7.9	6.8	0.8	-0.298
**Lys (K)**	6.1	5.7	5.2	6.1	5.1	3.3	5.2	1.0	6.3	8.9	4.4	4.3	4.9	8.1	6.2	2.0	-1.019
**Leu (L)**	10.3	10.4	10.1	10.7	10.1	10.4	10.3	0.2	10.5	11.2	10.7	11.4	10.8	9.6	10.7	0.6	**-1.370**
**Met (M)**	2.4	2.6	2.8	2.4	2.5	2.0	2.5	0.3	2.4	2.3	2.8	2.0	2.7	2.6	2.5	0.3	-0.137
**Asn (N)**	5.1	3.8	4.6	4.8	4.5	2.9	4.3	0.8	4.9	5.9	3.9	4.1	3.9	5.6	4.7	0.9	-0.883
**Pro (P)**	3.5	3.9	4.0	3.6	3.9	5.0	4.0	0.5	3.7	3.3	4.4	5.1	4.0	3.2	4.0	0.7	0.140
**Gln (Q)**	4.5	3.7	4.7	4.4	4.8	3.7	4.3	0.5	4.6	3.7	4.4	5.5	5.2	3.6	4.5	0.8	-0.602
**Arg (R)**	3.8	5.0	4.4	4.0	4.4	6.1	4.6	0.9	4.5	3.5	5.5	5.1	5.0	3.8	4.6	0.8	0.115
**Ser (S)**	7.2	6.6	6.6	6.7	6.9	6.8	6.8	0.2	5.8	6.8	5.8	5.9	6.3	6.1	6.1	0.4	**3.684**
**Thr (T)**	5.6	5.3	5.8	5.4	5.5	5.6	5.5	0.2	5.2	4.4	5.4	5.5	5.2	5.5	5.2	0.4	**1.779**
**Val (V)**	6.6	6.7	6.6	6.4	6.9	8.0	6.9	0.6	6.7	5.6	7.1	6.6	7.0	6.9	6.6	0.5	0.724
**Trp (W)**	1.1	1.0	1.2	1.1	1.2	1.5	1.2	0.2	1.1	0.7	1.5	1.6	1.3	1.0	1.2	0.3	-0.023
**Tyr (Y)**	3.1	3.0	3.1	3.1	3.1	2.1	2.9	0.4	3.1	3.7	2.8	2.9	3.0	4.0	3.3	0.5	-1.310
**Amino Acid Property Groups**																	
**Tiny**	28.3	29.4	29.1	28.1	29.0	33.8	29.6	2.1	27.0	24.9	29.2	28.0	28.4	25.1	27.1	1.8	**2.235**
**Small**	49.1	48.8	50.3	48.4	50.2	55.1	50.3	2.4	47.3	44.4	49.8	48.9	48.4	46.6	47.6	1.9	**2.166**
**aliphatic**	24.1	24.2	23.5	24.5	23.2	23.4	23.8	0.5	24.3	24.0	23.8	24.3	23.9	24.4	24.1	0.3	-1.157
**aromatic**	10.8	10.3	10.2	10.7	10.9	8.9	10.3	0.8	10.7	11.9	10.5	10.4	10.8	11.1	10.9	0.6	**-1.559**
**non polar**	54.2	56.5	55.0	55.2	55.0	58.9	55.8	1.7	55.1	53.1	57.3	56.6	55.9	52.9	55.1	1.8	0.615
**Polar**	45.8	43.5	45.0	44.8	45.0	41.1	44.2	1.7	44.9	46.9	42.7	43.4	44.1	47.1	44.8	1.8	-0.601
**charged**	23.4	24.1	23.3	23.4	23.4	22.2	23.3	0.6	24.3	26.1	23.1	22.4	23.5	26.2	24.3	1.6	**-1.414**
**Basic**	12.0	12.7	11.8	12.2	11.8	11.3	12.0	0.5	12.8	14.5	12.2	11.3	12.3	13.7	12.8	1.2	**-1.663**
**Acidic**	11.5	11.4	11.5	11.2	11.6	10.9	11.3	0.3	11.5	11.6	10.9	11.1	11.2	12.5	11.5	0.5	-0.541
**Neutral**	25.9	25.2	26.0	25.2	26.3	26.4	25.8	0.5	24.4	22.8	25.3	26.1	25.8	23.3	24.6	1.3	**2.057**
**hydrophilic**	30.8	29.6	30.3	30.6	30.4	26.9	29.8	1.5	31.8	33.6	29.2	30.2	30.2	33.7	31.4	1.9	**-1.696**
**hydrophobic**	44.3	45.0	44.4	45.1	44.2	45.4	44.7	0.5	44.7	44.0	45.5	44.2	45.2	43.5	44.5	0.8	0.527

The average (*Avg*) values among each set of proteomes along with their standard deviations (SD) are also given. Significant compositional differences as indicated by t-test parameter are shown in bold.

**^a^Psychrophiles (OGT < 20°C)**: *Colwellia psychrerythraea *34H (Abbreviation: Cpsy-P1), *Desulfotalea psychrophila LSv54 *(Dpsy-P2), *Psychrobacter cryohalolentis *K5 (Pcry-P3)*, Psychromonas ingrahamii 37 *(Ping-P4)*Pseudoalteromonas atlantica *T6c (Patl-P5) and *Renibacterium salmoninarum *ATCC 33209 (Rsal-P6).

**^b^Mesophiles (20°C < OGT < 45°C)**: *Haemophilus influenzae *(Hinf-M1), *Synechocystis PCC680 *(Spcc-M2), *Helicobacter pylori *26695 (Hpyl-M3)*, Escherichia coli *K12 (Ecol-M4)*, Vibrio cholerae *(Vcho-M5), *Lactobacillus salivarius *UCC11 (Lsal-M6).

### Amino acid composition preferences

The *t-test *results demonstrate significant preferences in frequencies of amino acid occurrences and property groups in psychrophilic proteomes as compared to mesophilic proteomes or *vice versa *(Table 1). The compositional trend of AA is somewhat similar in both types of genomes. However, as indicated by t-values from Table 1, there are a few AA residues such as A, D, S and T, significantly preferred in psychrophiles as compared to mesophiles. On the other hand, AA residues E and L are significantly less favored in psychrophile proteomes. When comparing frequencies of occurrences of property groups of AAs, we observe that tiny/small and neutral amino acid groups are significantly preferred in psychrophiles where as charged, basic, aromatic and hydrophilic groups are significantly less favored as shown by their corresponding t-values in Table 1. When we compared the AA compositions of the sequences in alignments of respective orthologous proteins alone (the data not given), we observed similar trends.

### Secondary Structural Elements

The composition of AA of psychrophilic and mesophilic proteomes in three major secondary structural elements, α-helices, β-sheets and coils, is given in Table 2. Collectively taken, the psychrophilic proteomes contain significantly less number of residues (~2%) in the α-helices and significantly more number of residues (~2%) in the coil regions. The majority of amino acids exhibit similar compositions in either of the two genome sequences. However, amino acids E, F L, N and Y show significantly low frequencies in α-helices of psychrophilic proteomes and amino acids A, D, G, S, T, and V are significantly high in the coil region of psychrophilic proteomes. The amino acid, E is significantly low in the coil region of psychrophilic proteomes. Except in an increase in Alanine residues, β-sheets of psychrophile proteomes did not show any significant changes as compared to mesophiles.

**Table 2:** Distribution of amino acids and property group parameters in the predicted secondary structural elements of psychrophilic and mesophilic proteomes.

	**α-Helix**					**β-Sheet**					**Coil**				
**Amino Acids**	**Psychro**		**Meso**			**Psychro**		**Meso**			**Psychro**		**Meso**		
	**Avg**	**SD**	**Avg**	**SD**	**t-val**	**Avg**	**SD**	**Avg**	**SD**	**t-val**	**Avg**	**SD**	**Avg**	**SD**	**t-val**
**Ala (A)**	5.23	0.95	4.79	0.09	0.906	1.15	0.07	1.05	0.12	**1.745**	2.83	0.61	2.27	0.56	**1.963**
**Cys (C)**	0.41	0.11	0.42	0.00	-0.097	0.22	0.05	0.23	0.04	-0.026	0.37	0.08	0.35	0.02	0.524
**Asp (D)**	1.90	0.13	1.88	0.00	0.176	0.52	0.09	0.50	0.02	0.466	3.09	0.23	2.75	0.34	**2.660**
**Glu (E)**	3.04	0.22	3.42	0.02	**-2.480**	0.70	0.09	0.74	0.04	-1.086	2.09	0.10	2.18	-0.08	**-1.487**
**Phe (F)**	2.03	0.24	2.24	0.01	**-1.378**	0.97	0.17	1.02	0.15	-0.526	1.07	0.07	1.10	-0.03	-0.544
**Gly (G)**	1.81	0.19	1.87	0.01	-0.593	0.66	0.08	0.68	0.05	-0.494	4.58	0.71	4.11	0.47	**1.392**
**His (H)**	0.83	0.08	0.83	0.00	0.047	0.36	0.04	0.35	0.03	0.366	0.92	0.07	0.91	0.01	0.295
**Ile (I)**	3.26	0.49	3.45	0.03	-0.722	1.97	0.29	1.99	0.20	-0.134	1.38	0.16	1.32	0.06	0.734
**Lys (K)**	2.48	0.54	3.00	0.17	-1.123	0.65	0.16	0.77	0.21	-1.043	2.11	0.36	2.41	-0.30	-0.876
**Leu (L)**	6.03	0.23	6.42	0.01	**-2.619**	2.01	0.14	2.04	0.19	-0.294	2.29	0.13	2.24	0.06	0.612
**Met (M)**	1.22	0.16	1.26	0.01	-0.363	0.38	0.05	0.38	0.05	-0.245	0.86	0.05	0.84	0.02	0.633
**Asn (N)**	1.36	0.27	1.59	0.02	**-1.383**	0.41	0.12	0.45	0.07	-0.725	2.51	0.46	2.67	-0.16	-0.572
**Pro (P)**	0.98	0.07	1.01	0.01	-0.409	0.26	0.03	0.28	0.04	-0.950	2.76	0.51	2.65	0.11	0.353
**Gln (Q)**	2.27	0.21	2.45	0.03	-0.956	0.53	0.10	0.54	0.08	-0.359	1.51	0.18	1.53	-0.02	-0.166
**Arg (R)**	2.31	0.44	2.32	0.03	-0.041	0.70	0.06	0.71	0.12	-0.175	1.61	0.37	1.53	0.07	0.383
**Ser (S)**	2.65	0.17	2.55	0.01	0.798	0.90	0.14	0.81	0.10	1.190	3.26	0.22	2.76	0.50	**4.981**
**Thr (T)**	2.07	0.11	2.06	0.01	0.133	1.15	0.09	1.07	0.10	1.335	2.32	0.20	2.06	0.25	**2.418**
**Val (V)**	2.98	0.25	2.97	0.01	0.038	2.34	0.20	2.28	0.15	0.598	1.56	0.19	1.38	0.18	**1.912**
**Trp (W)**	0.62	0.09	0.66	0.01	-0.431	0.25	0.04	0.24	0.07	0.186	0.32	0.06	0.30	0.02	0.598
**Tyr (Y)**	1.32	0.19	1.51	0.01	**-1.554**	0.75	0.15	0.83	0.11	-1.066	0.86	0.09	0.92	-0.06	-0.979
**All AA**	44.81	1.56	46.73	0.18	**-2.507**	16.88	1.58	16.98	0.34	-0.153	38.31	1.90	36.29	2.02	**2.379**
**Amino Acid Property Groups**															
**Tiny**	12.18	0.90	11.70	0.14	0.913	4.08	0.36	3.84	0.24	**1.336**	13.37	1.54	11.56	0.11	**2.537**
**Small**	19.39	0.95	19.16	0.18	0.415	7.62	0.58	7.37	0.31	0.946	23.29	2.03	21.02	0.18	**2.435**
**Aliphatic**	12.27	0.56	12.85	0.01	**-2.279**	6.33	0.31	6.32	0.15	0.090	5.23	0.22	4.94	0.01	**2.443**
**Non-polar**	25.90	1.07	26.62	0.14	-1.241	10.97	0.80	11.03	0.28	-0.171	18.89	1.80	17.49	0.24	**1.594**
**Aromatic**	4.81	0.39	5.24	0.01	**-2.235**	2.32	0.36	2.44	0.17	-0.719	3.18	0.17	3.24	0.00	-0.651
**Polar**	18.91	0.95	20.11	0.21	**-1.987**	5.91	0.78	5.95	0.14	-0.129	19.42	0.83	18.80	0.09	1.340
**Charged**	10.56	0.57	11.46	0.17	**-1.923**	2.93	0.35	3.06	0.14	-0.895	9.82	0.16	9.78	0.05	0.192
**Basic**	5.61	0.36	6.15	0.06	**-1.863**	1.71	0.17	1.82	0.11	-1.353	4.64	0.07	4.85	0.04	-1.041
**Acidic**	4.94	0.22	5.31	0.03	**-1.892**	1.22	0.18	1.24	0.03	-0.335	5.19	0.21	4.93	0.01	**2.287**
**Neutral**	9.64	0.36	9.77	0.05	-0.469	3.59	0.43	3.46	0.16	0.674	12.59	0.98	11.38	0.09	**2.430**
**H-philic**	13.36	0.69	14.67	0.18	**-2.552**	3.51	0.52	3.72	0.13	-0.940	12.91	0.67	13.06	0.11	-0.356
**H-phobic**	23.11	0.85	23.73	0.09	**-1.352**	10.04	0.71	10.06	0.22	-0.067	11.56	0.66	10.72	0.02	**2.715**

Significant compositional differences as indicated by t-test parameter are shown in bold.

When evaluated for the frequencies of occurrence of property groups of amino acids, the majority of them show significantly low frequencies in helices of psychrophilic proteomes. Except in tiny group of AAs, the β-sheet regions of psychrophile proteomes did not show any significant changes (Table 2). The tiny, small, hydrophobic, neutral, acidic, aliphatic, and non-polar amino acid groups showed significantly high frequencies in the coil/loop regions of psychrophilic proteomes.

### Comparative Proteome Analysis

Towards identification of residue substitutions, likely to have undergone in psychrophilic proteins as the species adapted to cold temperatures, a comparative proteome analysis was performed on the basis of amino acid substitutions occurred between the orthologous protein sequences of psychrophile and mesophile proteomes. The orthologous sequence pairs for a protein sequence with significant length coverage only were considered for the analysis. Coverage of hits with respect to each proteome is shown in Table 3. On average 16.3% psychrophile proteins have orthologous proteins in mesophiles we used. This was cross-checked using mesophile proteomes as query sequences and the psychrophile proteomes as subject sequences, where we found 13.9% orthologs (Table 3). We used sequence alignments of these sequence pairs to compute substitutions of amino acids between mesophilic and psychrophilic proteomes and *vice versa *and the obtained values were averaged. On average, ten to twenty five percent of sequences from individual proteomes exhibited best hit homologues from members of other thermal groups (Table 3). This percent depended on the size of the proteome under consideration. The higher the number of proteins in a query proteome the higher percentage of hits from the subject proteomes searched. This may be because some of the paralogous sequences selecting the same protein as its ortholog (this redundancy was removed in our final data). We also considered homologous proteins among the psychrophiles and mesophiles, and calculated the substitutions within them to use as background substitution frequencies [see Eqn. (ii) and (iii)]. We observed, on average 16.7% and 17.1% orthologous proteins within the psychrophilic and mesophilic proteomes, respectively (Table 3).

**Table 3:** Percentage of orthologs (best hits) in Psychrophiles and Mesophiles with respect to total number of sequences (size) in each of organisms considered.

	**Psychrophiles**							**Mesophiles**							**Size**
**Genome**	**Cpsy**	**Dpsy**	**Pcry**	**Ping**	**Patl**	**Rsal**	**AVG**	**Hinf**	**Spcc**	**Hpyl**	**Ecol**	**Vcho**	**Lsal**	**AVG**	
**Cpsy**	100.0	12.5	17.8	15.5	14.7	9.5	**14.0**	21.1	10.4	15.3	12.2	14.1	13.0	**14.4**	3545
**Dpsy**	10.4	100	17.6	14.5	11.4	9.5	**12.7**	22.8	12.1	20.1	12.3	13.3	15.5	**16.0**	3507
**Pcry**	13.2	15.5	100	17.9	15.3	12.6	**14.9**	28.8	13.7	22.3	15.0	16.3	17.4	**18.9**	4910
**Ping**	13.8	15.3	21.0	100	15.2	11.4	**15.3**	28.0	12.6	19.6	15.3	17.8	17.4	**18.5**	3234
**Patl**	14.5	13.4	20.1	16.9	100	10.6	**15.1**	23.7	11.7	16.7	13.9	15.9	14.8	**16.1**	2511
**Rsal**	9.0	10.9	15.4	12.5	10.0	100	**11.6**	20.3	11.2	14.6	11.3	11.3	16.8	**14.3**	4281
**AVG**	**12.2**	**13.5**	**18.4**	**15.5**	**13.3**	**10.7**	**16.7**	**24.1**	**11.9**	**18.1**	**13.3**	**14.8**	**15.8**	**16.3**	**3665**
**Hinf**	14.2	17.6	24.7	20.9	16.4	13.6	**17.9**	100	14.4	27.6	19.5	20.3	21.2	**20.6**	1657
**Spcc**	8.3	12.0	15.4	11.7	9.7	9.7	**11.1**	18.4	100	16.6	9.9	10.2	14.0	**13.7**	3569
**Hpyl**	9.6	14.4	18.1	13.7	10.7	10.0	**12.7**	25.4	11.7	100	11.7	12.5	16.4	**15.5**	1576
**Ecol**	11.6	14.2	19.5	16.8	13.4	11.7	**14.5**	29.7	11.8	18.7	100	16.6	17.7	**18.9**	4243
**Vcho**	12.8	14.4	19.4	17.8	14.6	10.5	**14.9**	27.1	11.6	18.9	14.9	100	16.0	**17.7**	3835
**Lsal**	8.9	12.8	16.0	13.0	10.1	12.2	**12.2**	23.7	11.4	19.4	11.8	11.9	100	**15.6**	2017
**AVG**	**10.9**	**14.2**	**18.8**	**15.6**	**12.5**	**11.3**	**13.9**	**24.9**	**12.2**	**20.1**	**13.5**	**14.3**	**17.1**	**17.0**	**2816**

### Amino Acid Substitution Patterns

Log odd scores (LOS) of AA substitutions were calculated using the frequency of occurrence of substitutions among orthologous proteins of psychrophiles and mesophiles by normalizing with frequency of occurrence of substitutions within the proteomes of the same temperature sensitive group. In LOS calculation the substitutions influenced by factors other than temperature are nullified and values represent true substitution due to cold adaptation of species. In Table 4 we show LOS_Meso _values computed using equation (ii) as described in methods. These values clearly show that psychrophilic proteins avoid containing the amino acids E, F, K, N and Y. On the other hand they prefer containing the residues A, D, G, S, and T as compared to mesophile proteins. The individual values in Table 4 show that to what extant certain substitutions are favored or avoided depending on corresponding LOS_Meso _score +ve or -ve, respectively. For example the W in mesophiles mutating to S in the psychrophiles is highly favored with LOS scores of 10.9. On the other hand G in mesophiles mutating to K in psychrophiles is avoided with LOS score of -12.4. The LOS_Psychro _[Eqn. (iii)] scores calculated using substitution frequencies among psychrophiles as normalizing factor have shown similar results (data not shown).

**Table 4:** Log odd scores (LOS) of amino acid substitutions calculated using the equation (ii).

**Psychrophiles**																					
		**A**	**C**	**D**	**E**	**F**	**G**	**H**	**I**	**K**	**L**	**M**	**N**	**P**	**Q**	**R**	**S**	**T**	**V**	**W**	**Y**
**Mesophiles**	**A**	2.0	-2.5	3.4	**-5.1**	*-4.6*	1.4	-0.6	-1.3	**-11.2**	-2.5	-2.6	**-6.4**	-1.6	-2.9	-0.3	1.3	0.8	-0.4	0.8	**-7.7**
	**C**	1.8	1.2	1.2	-3.2	-3.4	1.7	**6.1**	-2.0	**-9.4**	-2.1	-4.2	-4.3	-1.5	9.3	2.3	1.3	-0.8	-0.2	1.5	*-6.9*
	**D**	**8.2**	2.9	1.1	**-5.1**	**-5.2**	**4.1**	-3.0	2.0	**-9.5**	-1.1	-0.6	**-6.0**	-2.9	-2.7	2.0	**3.8**	2.0	**4.1**	1.7	**-6.5**
	**E**	**7.5**	1.1	**3.2**	**-2.4**	**-6.7**	**4.9**	0.2	1.5	**-8.9**	-1.9	-1.6	**-4.6**	-3.3	-1.4	0.8	**5.4**	**4.4**	**3.5**	-4.9	**-7.2**
	**F**	**4.7**	-0.2	*5.5*	-4.5	-0.1	3.2	**3.4**	0.0	**-8.7**	-0.6	0.4	-2.7	0.8	-0.8	3.0	**5.0**	2.4	1.4	0.0	**-3.2**
	**G**	**3.1**	0.4	2.2	**-4.6**	-3.9	0.8	**-8.8**	-1.7	**-12.4**	-3.5	-2.0	**-9.3**	-0.9	-4.0	-1.9	1.6	0.9	2.5	1.7	**-8.3**
	**H**	**7.5**	0.6	2.9	**-4.6**	**-6.2**	4.0	0.4	-1.7	**-8.2**	-1.0	-2.0	**-4.0**	-2.0	-1.6	0.7	**5.8**	1.4	0.9	0.1	**-7.4**
	**I**	**4.8**	-0.9	*6.1*	**-6.5**	-2.7	3.5	3.3	-1.1	**-7.9**	-1.6	0.2	-2.6	0.0	0.7	1.0	**4.8**	*2.8*	1.2	-4.4	**-5.7**
	**K**	**6.6**	2.7	4.8	-4.8	-5.3	4.4	-3.4	2.4	**-3.7**	0.0	-1.3	-3.7	-2.6	-3.1	-0.2	**7.0**	**5.2**	3.5	-3.1	**-8.8**
	**L**	**5.4**	1.1	3.4	**-5.8**	**-3.1**	2.6	**4.3**	-0.3	**-8.5**	-0.2	0.8	-3.9	-0.2	0.7	1.5	**4.9**	2.4	1.4	-3.3	**-4.7**
	**M**	**3.7**	-3.2	*3.9*	**-6.2**	**-4.2**	3.7	1.3	-2.5	**-8.7**	-2.2	0.6	-2.5	-0.1	1.3	1.0	*3.2*	1.3	0.7	-1.7	**-6.2**
	**N**	**6.6**	2.9	**4.0**	**-3.6**	-3.7	3.6	-3.0	0.5	**-7.5**	0.1	-1.5	-1.9	-2.4	-2.9	1.4	**5.4**	2.6	3.2	-2.3	**-4.6**
	**P**	**6.7**	0.4	**4.6**	**-5.4**	**-6.7**	**4.4**	0.0	-1.6	**-10.5**	-3.0	1.7	**-5.7**	0.2	0.9	-0.1	**4.1**	3.1	1.0	-1.6	**-11.0**
	**Q**	**8.2**	5.0	**5.0**	**-4.2**	**-5.4**	**6.0**	-0.8	1.3	**-7.2**	-0.3	-0.1	-4.0	0.3	-0.5	1.1	**6.0**	**5.2**	**4.2**	0.1	**-7.0**
	**R**	**6.8**	2.9	*5.0*	*-5.4*	-5.2	**5.4**	-2.4	-0.7	**-6.8**	-2.2	-2.8	-2.5	0.4	-1.8	0.5	**6.1**	*5.0*	2.1	-2.5	**-7.8**
	**S**	**3.1**	-0.7	**2.4**	**-4.8**	-2.8	**3.1**	-0.6	0.8	**-8.0**	-1.2	-1.1	**-4.1**	-0.5	-2.6	0.1	1.5	0.6	2.4	0.6	**-5.4**
	**T**	**3.9**	0.0	2.8	**-4.1**	-3.5	**3.8**	-0.7	-1.5	**-9.3**	-1.0	-0.7	**-3.3**	0.4	-0.6	1.6	**2.6**	0.7	1.3	-3.2	**-7.6**
	**V**	**2.6**	0.1	**3.6**	**-5.4**	*-3.7*	*4.4*	1.1	-1.8	**-9.3**	-2.1	-0.1	-2.9	-0.4	0.5	2.1	*3.6*	*1.8*	0.3	-0.6	**-8.1**
	**W**	*9.6*	5.4	5.0	-4.9	-2.8	3.3	2.9	-1.1	**-11.1**	0.5	1.1	-4.2	0.4	4.3	1.8	**10.9**	1.0	2.4	3.3	-4.1
	**Y**	**5.8**	0.6	*4.2*	-2.8	-0.3	4.1	**3.9**	0.3	**-7.2**	-0.7	0.5	**-3.4**	-0.9	-0.1	3.4	**7.6**	3.0	2.3	-0.4	-1.2
	**All**	**3.8**	0.3	**2.5**	**-3.8**	-1.9	1.8	-0.2	-0.9	**-6.9**	-0.9	0.0	**-3.7**	-0.4	-1.3	0.6	**3.2**	1.8	1.0	0.7	**-3.8**

Student t-test was further applied to evaluate level of significance of LOS substitution scores and these data were shown in Additional files (Additional file 1 &2). Table Additional file 1(a) shows t-values for the LOS_Meso _scores and Additional file 1(b) shows t-values for the LOS_Psychro _scores. The LOS_Meso _scores (Table 4) with their corresponding t-values (Additional file 1) greater than 1.37 or less than -1.37 could be considered as significantly preferred or avoided, respectively, at 90% confidence level (shaded in color, Table 4). It can be seen from the table that there are about 45% substitutions that are shown to be significant. Further, the LOS_Meso _for AA property group substitutions are given in Table 5 and their corresponding t-values are given in Additional file 2. It is clearly seen from Table 5 that there is high preference for tiny, small and neutral AAs whereas charged (including both basic and acidic) aromatic and hydrophilic AAs are avoided significantly in the psychrophiles as compared to mesophiles.

**Table 5:** Log odd scores (LOS) of amino acid substitutions calculated using the Eqn (ii).

		**Psychrophiles**											
		**T**	**S**	**Al**	**Ar**	**Np**	**P**	**C**	**B**	**A**	**N**	**Hl**	**Hb**
**Mesophiles**	**Tiny (T)**	1.47	1.12	-0.68	-3.86	0.79	-0.94	-3.31	-5.40	-1.32	0.87	-3.67	0.74
	**Small (S)**	1.89	1.12	-0.59	-3.93	0.58	-0.86	-2.03	-4.96	-0.48	1.11	-2.41	0.44
	**Aliphatic (Al)**	3.34	1.60	-0.40	-3.19	-0.29	-0.10	-2.60	-2.66	-2.50	2.69	-2.52	-0.33
	**Aromatic(Ar)**	4.67	2.75	0.05	-0.40	-0.02	0.12	-0.41	-0.32	-0.73	1.94	-2.05	-0.11
	**Nonpolar (Np)**	2.01	1.31	-0.49	-1.49	0.07	-1.01	-2.91	-3.93	-1.61	1.36	-3.31	-0.08
	**Polar (P)**	3.50	2.03	0.59	-2.66	1.65	-0.84	-1.66	-2.55	-0.76	1.44	-1.91	1.51
	**Charged (C)**	5.52	2.49	0.68	-2.40	1.69	-1.13	-1.54	-2.24	-0.83	1.69	-1.87	1.69
	**Basic (B)**	5.75	3.44	0.47	-2.07	1.15	-1.16	-1.72	-1.76	-1.50	1.56	-2.07	0.89
	**Acidic (A)**	5.29	1.94	1.00	-3.59	2.39	-1.10	-1.36	-4.84	-0.70	1.86	-1.68	2.84
	**Neutral (N)**	1.78	1.39	0.16	-2.12	1.02	-0.69	-2.26	-3.11	-1.10	0.95	-2.68	0.98
	**H-philic (Hi)**	5.53	2.41	0.97	-3.90	2.14	-1.07	-1.64	-2.66	-0.72	1.76	-1.78	2.17
	**H-phobic (Hb)**	2.38	1.55	-0.48	-1.32	-0.08	-0.57	-2.57	-2.98	-1.98	1.97	-2.90	-0.14
	**All**	2.56	1.60	-0.34	-1.89	0.42	-0.91	-1.83	-2.79	-0.87	1.34	-2.16	0.25

The +ve values indicates that the corresponding substitution of mesophilic amino acid property groups to the respective psychrophilic groups is higher than such substitutions within the mesophilic homologous proteins. The values shown to be significant at 90% confidence level by student t-test are shown in bold.

## Discussion

Our objective in this study was to analyze systematically the compositional variation and substitution preferences of amino acids in proteomes of psychrophiles compared to the proteomes of mesophiles to investigate general proteome wide characteristics for cold adaptation. We considered total compositional differences in proteomes as well as compositional differences in their orthologous proteins alone. We performed analysis at different levels, through simple amino acid compositions, student t-test and finally by substitution patterns in their orthologous proteins. Some of the methods we used were previously applied [26,29-31] but not to the complete proteome analysis.

In psychrophiles individual residue compositions show that there is a significant preference for A, D, S and T content and significant avoidance of E and L content and moderate preference for G and avoidance for F and K content (Table 1). All these residue preferences and avoidance directly show a strong correlation with respect to avoidance for helical content in psychrophiles, as S, D and G are helix breakers [32] and T is a helix indifferent. Likewise, the presence of E tends to favor formation of helical structures and L tends to stabilize helical structures [32] that are highly avoided in psychrophiles. Amino acid D is observed to be unstable at high temperatures and therefore its frequency observed to decrease as optimal growth temperature of organisms increase [33]. Reverse trends are observed for E to counter the trend in favor of making ion pair interactions to form salt-bridges at higher temperatures [34,35]. Helix destabilizing beta-branched residues (I, T and V) are preferred in beta sheets and loop regions of psychrophilic proteins [36,37]. The substitution pattern in the orthologous proteins of two temperature groups show several interesting features that are not readily seen in the simple AA compositions. On other hand, they strongly support observed differences in compositions apart from giving additional insights, such as what specific substitutions were more favored or avoided as shown by LOS values.

### Nonpolar Amino Acids

Our results in this study confirm that overall composition of nonpolar AA group did not show much difference in proteins belonging to psychrophiles and mesophiles, but there is a decrease in nonpolar AA group frequency in helices and a significant increase in loop regions among proteins of psychrophiles. This is in accordance with earlier findings that there are more nonpolar amino acids on the exposed surface area of the majority of psychrophilic proteins [35,38] as more loops are observed on surface regions. Among nonpolar residues, I, L and V belong to aliphatic group of residues, which are significantly reduced to favor protein flexibility. It has been widely accepted that the aliphatic amino acids would contribute to the hydrophobic interaction for maintaining conformational stability and rigidity in core region of the proteins [36,39,40]. Observed low average hydropathy and low aliphatic residues in psychrophiles are mainly contributed by significantly low in L composition [35].

### Tiny and Small Amino acids

Tiny and small amino acids are those with short side chains and are unable to participate in long range interactions among secondary structural elements and are usually confined to form local interactions. Overall their compositions are significantly increased in beta sheets and loops of psychrophilic proteins over mesophilic counter parts. This is also clear from substitutions observed in orthologous proteins. The amino acid G is devoid of side chain, is more flexible with greater rotational freedom, is capable of making cavities in the core parts of the proteins structures [39] and was shown to be in less frequency in thermophiles [41]. Whereas P with pyrolidine ring structure has restricted conformations and was shown to occur in higher frequency in thermophiles [40]. Our LOS values confirm that the amino acid G is preferred and the P is avoided in psychrophiles as compared to mesophiles.

### Charged amino acids

Charged residues are polar and hydrophilic. They contribute to ion pair electrostatic interactions that are important binding force for maintaining conformational stability in surface of the proteins [37,39,42]. The more charged residues were found in thermophilic proteins than in mesophilic proteins [36,40]. The charged AA (especially basic and hydrophilic) residues are significantly avoided in psychrophiles (Table 5). Present analysis also supports the notion that these residues are significantly avoided in psychrophiles as observed from compositions as well as LOS scores. The most striking feature of psychrophilic proteins is an increase in amino acid D and decrease in amino acid E over mesophiles. The charged residues in mesophiles are mainly replaced with small and tiny residues in psychrophiles.

### Aromatic Amino acids

Psychrophilic proteins and their secondary structural elements show significant decrease in aromatic amino acids. Especially F and Y that are capable of binding to cationic amino acid side chains of K and R in forming cation-π interactions and play important role in stabilizing three dimensional structure of proteins [43-45]. Our studies show that, a significant decrease in aromatic residues occurs by substitution with tiny/small and neutral amino acids in psychrophiles (Table 5). Finally, our LOS values, combining with PAM or BLOSUM mutual substitution scores, assist in selecting suitable mutations in designing mesophilic proteins to optimally function in cold temperatures or *vice versa*. For example substitution of E in mesophilic proteins with D may not change chemical characteristics significantly but may result in optimizing protein function in cold temperatures. In similar lines we observe that amino acid K is highly avoided and R is preferred in psychrophiles although both being basic amino acids.

## Conclusion

We analyzed compositions of individual amino acid residues, amino acid groups and their distribution pattern in secondary structures and then computed and quantified their substitution patterns and directional preferences between mesophiles and psychrophiles. Significant differences in composition of amino acid residues were observed between the mesophilic and psychrophilic proteins as summarized below: (i) we observed an increase in frequency of individual amino acids like A, D, S and T that avoid helices and a decrease in E and L amino acids; (ii) There is an increase in small/tiny and neutral group residues which contribute to protein flexibility and a decrease in charged amino acids, particularly basic as well as hydrophilic residues that contribute to ionic interactions; (iii) there is a decrease in aromatic amino acids residues that contribute to the cation-π interactions; (iv) there is a decrease in aliphatic residues which provide good covering and masking to produce hydrophobic pockets that are involved in stabilizing protein structure; (v) there is a reduction in amino acid preferences for helices and an increase of coil forming residues; (vi) we also observed a significant level of substitutions of aliphatic and charged amino acids by tiny/small or neutral amino acids; (vii) the results from this analysis, especially significant t-values of LOS substitution pairs, can be used as a knowledge base in rational design of mutations for engineering of mesophilic proteins to function optimally in cold temperatures or *vice versa*.

## Methods

Proteome sequences from six members each of available completely sequenced species of psychrophiles and mesophiles (listed in the foot note of Table 1) are collected. They were selected randomly from independent genuses of mesophiles and 6 of 9 available completely sequenced genomes of psychrophiles to control plausible variations from phylogenetic non-independence (PNI) as related species may share the similar traits due to shared ancestry. These proteome sequences were downloaded from the NCBI proteome project server in the fasta format. The growth temperatures of these species were obtained from NCBI [46] and/or PGTdb (Prokaryotic Growth Temperature database) [47]. We computed the frequencies of amino acid residues in the protein sequences of psychrophilic and mesophilic proteomes. We also grouped the amino acids into 12 property groups [48] as follows: Acidic amino acids group include D and E; aliphatic: I, L and V; aromatic: H, F, W and Y; basic: R, H, and K; charged: R, D, E, H and K; hydrophilic: D, E, K, N, Q and R; hydrophobic: A, C, F, I, L, M, V, W and Y neutral: G, Q, H, S and T; non-polar: A, C, G, I, L, M, F, P, V, W and Y; polar: R, N, D, E, Q, H, K, S and T; small: A, C, D, G, N, P, S, T, V and tiny: A, C, G, S and T. The sum of frequencies of amino acids that fall in each property group are calculated for psychrophiles and mesophiles and are also compared (Table 1). Some of the amino acids are included in more than one property group.

### Student *t-test*

To compare the means of two groups of data, *t-test *is essentially a good tool for the signal-to-noise metaphor used in research analysis. IIn present analysis, we compared the mean frequencies of single amino acids, 12 different property groups of amino acids and three secondary structural elements from all protein sequences of psychrophilic and mesophilic proteomes considered in this analysis. The *t*-values are calculated as follows:

(i) $$t=\frac{F_{Psychro}-F_{Meso}}{\sqrt{(Var_{Psychro}/n_{Psychro})+(Var_{Meso}/n_{Meso})}}$$

Where *Var*_*Psychro *_and *Var*_*Meso *_are the variance of residues or property groups; (*F*_*Psychro*_) and (*F*_*Meso*_) are mean frequencies of psychrophilic and mesophilic proteomes respectively. The *n*_*Psychro *_and *n*_*Meso *_are the total number of psychrophilic and mesophilic proteomes investigated in this study, respectively. Based on student's *t-distribution *table of significance, critical values for such t-test at various probabilities are as follows (see Table 6):

**Table 6:** t-test critical values

Probability	10%	5%	2.5%	2%	1%	0.5%
Critical Value	1.372	1.812	2.228	2.359	2.764	3.169

If *t*-value is positive and greater than critical value at 10% probability (1.372) then the mean frequency (*F*_*Psychro*_) of psychrophilic proteomes is significantly greater than that of the mesophilic proteomes (*F*_*Meso*_) at 90% or higher confidence level. If the frequency of residue or property group *t*-value is negative and less than -1.372 then the mean frequency of psychrophilic proteomes (*F*_*Psychro*_) is significantly less than that of mesophilic proteomes (*F*_*Meso*_) at 90% or higher confidence level [18,40,49].

### Secondary Structure Prediction

We predicted secondary structural elements in protein sequences using GTOP [50] and/or PSIPRED [51]. We used these predictions to compute frequencies of different amino acids and property groups of residues in three major secondary structural regions, helix (H), strand (E) and coil (C). PSIPRED is a highly reliable secondary structure prediction method with ~83% reported prediction accuracy. We have also tested its prediction accuracy on some of the known psychrophilic and mesophilic proteins to see if there is any significant difference in its prediction for psychrophilic proteins. A total of about 25 proteins, approximately 5000 residues, from each we observed that PSIPRED prediction accuracy was 78.42% and 80.89% for psychrophilic and mesophilic proteins, respectively.

### Comparative Analysis of Amino Acid Substitutions

All protein sequences from each mesophilic species in dataset were searched against each proteome of psychrophylic species and *vice versa*, using BLASTP [52] with 10^-3 ^expectation value cutoff and considerable length coverage. We picked up pair-wise alignments obtained from BLAST results of each protein sequence in a query proteome that showed best hit homolog (ortholog) in the subject proteome. The pair-wise alignments (exclusion of gapped regions) were parsed to calculate amino acid substitution counts between the two proteins from respective proteomes. The substitution counts were normalized to total amino acids present in their respective proteomes pairs individually and finally to all the pairs. The resultant frequency of substitutions was further used to calculate two types of likelihood log odd scores (*LOS*):

(ii) $$LOS_{Meso}=Log\frac{F(X_{Meso}\to Y_{Psychro})}{F(Y_{Meso}\to X_{Meso})}$$

(iii) $$LOS_{Psychro}=Log\frac{F(X_{Psychro}\to Y_{Meso})}{F(X_{Psychro}\to Y_{Psychro})}$$

Where *F*(*X*_*Meso *_→ *Y*_*Psychro*_) represent normalized frequency of amino acid *X *in mesophile substituted by an amino acid *Y *in psychrophile. The *LOS *values are calculated by using background substitution frequencies among the mesophilic and/or psychrophilic proteomes in the denominator. The *LOS *scores, therefore, indicated the pattern of substitutions that are predominantly due to their thermal adaptation and therefore minimize the effect of substitutions due to any speciation events in the evolution process.

## Authors' contributions

MRPR and BVBR conceived of the study and participated in its design and coordination. MRPR carried out the analysis and authored the first draft of this manuscript. BVBR was involved in useful discussions and provided comments and revisions to the final version of this text. Both authors read and approved the final manuscript.

## Supplementary Material

Additional file 1 **T-test values for amino acid substitution preferences between proteins of Mesophilic and Psychrophilic proteomes**. The t-values calculated using LOS substitution scores of mutation frequencies normalized with substitution frequencies within the (a) mesophiles (m2m) and (b) psychrophiles (p2p). Click here for file

Additional file 2 **T-test values for amino acid property groups' substitution preferences between proteins of Mesophilic and Psychrophilic proteomes**. The t-values calculated using LOS substitution scores of mutation frequencies normalized with substitution frequencies within the mesophiles Click here for file
