# Supplementary material for: Comparative proteome analysis of psychrophilic versus mesophilic bacterial species: Insights into the molecular basis of cold adaptation of proteins
Source: BMC Genomics. 2009 Jan 8;10:11. doi: 10.1186/1471-2164-10-11 (PMC2653534; doi:10.1186/1471-2164-10-11)
Supplement: Additional file 1 — T-test values for amino acid substitution preferences between proteins of Mesophilic and Psychrophilic proteomes. The t-values calculated using LOS substitution scores of mutation frequencies normalized with substitution frequencies within the (a) mesophiles (m2m) and (b) psychrophiles (p2p). [file 1471-2164-10-11-S1.doc]

| **Additional file 1**: t-values for amino acid substitution preferences between proteins of Mesophilic and Psychrophilic proteomes. | | | | | | | | | | | | | | | | | | | | | |
| --- | --- | --- | --- | --- | --- | --- | --- | --- | --- | --- | --- | --- | --- | --- | --- | --- | --- | --- | --- | --- | --- |
| **a)** | **m2p/m2m** | | | | **Psychrophiles** | | | | | | | | | | | | | | | | |
|  |  | **A** | **C** | **D** | **E** | **F** | **G** | **H** | **I** | **K** | **L** | **M** | **N** | **P** | **Q** | **R** | **S** | **T** | **V** | **W** | **Y** |
| **Mesophiles** | **A** | 1.08 | -0.90 | 1.24 | -1.93 | -1.59 | 0.83 | -0.24 | -0.51 | -2.54 | -1.04 | -1.12 | -2.06 | -0.93 | -1.12 | -0.11 | 0.65 | 0.44 | -0.29 | 0.26 | -2.33 |
|  | **C** | 0.48 | 0.28 | 0.23 | -0.74 | -0.99 | 0.54 | 1.69 | -0.53 | -1.81 | -0.70 | -1.09 | -1.03 | -0.32 | 1.27 | 0.59 | 0.42 | -0.22 | -0.05 | 0.25 | -1.95 |
|  | **D** | 2.99 | 0.52 | 1.03 | -3.06 | -1.59 | 1.60 | -1.22 | 0.64 | -2.42 | -0.36 | -0.17 | -2.81 | -0.96 | -1.07 | 0.56 | 2.32 | 1.08 | 1.70 | 0.46 | -2.18 |
|  | **E** | 2.88 | 0.24 | 2.17 | -1.91 | -2.10 | 1.87 | 0.08 | 0.52 | -2.55 | -0.74 | -0.61 | -1.98 | -1.07 | -0.74 | 0.23 | 4.01 | 2.82 | 1.87 | -1.18 | -2.49 |
|  | **F** | 1.43 | -0.05 | 1.40 | -1.21 | -0.06 | 0.89 | 1.46 | 0.02 | -2.04 | -0.34 | 0.16 | -0.76 | 0.20 | -0.21 | 0.74 | 2.08 | 0.90 | 0.51 | 0.01 | -2.04 |
|  | **G** | 1.68 | 0.13 | 0.95 | -1.80 | -1.13 | 1.11 | -2.99 | -0.55 | -2.60 | -1.32 | -0.70 | -2.68 | -0.36 | -1.53 | -0.64 | 0.76 | 0.39 | 1.01 | 0.62 | -2.42 |
|  | **H** | 2.75 | 0.15 | 1.35 | -2.21 | -2.68 | 1.30 | 0.22 | -0.62 | -2.78 | -0.47 | -0.69 | -1.82 | -0.67 | -0.71 | 0.28 | 3.58 | 0.61 | 0.40 | 0.05 | -3.04 |
|  | **I** | 1.78 | -0.25 | 1.96 | -2.45 | -1.25 | 1.30 | 1.22 | -0.76 | -2.28 | -0.95 | 0.10 | -0.98 | 0.01 | 0.29 | 0.29 | 2.32 | 1.47 | 0.64 | -1.38 | -2.06 |
|  | **K** | 1.37 | 0.52 | 1.10 | -1.18 | -1.21 | 0.83 | -1.02 | 0.59 | -1.55 | -0.01 | -0.31 | -1.02 | -0.46 | -0.77 | -0.05 | 1.96 | 1.42 | 0.89 | -0.60 | -2.17 |
|  | **L** | 1.94 | 0.36 | 1.03 | -1.95 | -1.64 | 0.92 | 2.09 | -0.16 | -2.40 | -0.16 | 0.43 | -1.33 | -0.06 | 0.31 | 0.44 | 2.75 | 1.16 | 0.65 | -1.16 | -1.77 |
|  | **M** | 1.51 | -0.85 | 1.45 | -2.45 | -1.68 | 1.23 | 0.51 | -1.21 | -2.43 | -1.24 | 0.16 | -1.00 | -0.03 | 0.67 | 0.32 | 1.47 | 0.71 | 0.35 | -0.60 | -2.02 |
|  | **N** | 2.09 | 0.67 | 1.94 | -1.45 | -1.21 | 1.00 | -1.26 | 0.20 | -2.31 | 0.03 | -0.52 | -1.09 | -0.67 | -1.01 | 0.39 | 3.37 | 1.26 | 1.26 | -0.52 | -1.71 |
|  | **P** | 3.53 | 0.07 | 1.44 | -1.71 | -1.60 | 1.59 | 0.00 | -0.48 | -1.97 | -0.93 | 0.50 | -1.55 | 0.16 | 0.34 | -0.04 | 1.71 | 1.08 | 0.42 | -0.41 | -2.42 |
|  | **Q** | 2.95 | 1.29 | 1.89 | -1.76 | -1.48 | 2.15 | -0.34 | 0.44 | -1.78 | -0.11 | -0.04 | -1.21 | 0.13 | -0.17 | 0.36 | 2.53 | 2.29 | 1.82 | 0.02 | -2.33 |
|  | **R** | 2.10 | 0.64 | 1.43 | -1.57 | -1.25 | 1.77 | -0.99 | -0.20 | -1.68 | -0.70 | -0.99 | -0.76 | 0.14 | -0.61 | 0.23 | 2.25 | 1.82 | 0.69 | -0.70 | -1.93 |
|  | **S** | 1.54 | -0.25 | 1.42 | -3.13 | -1.30 | 1.50 | -0.38 | 0.33 | -2.58 | -0.69 | -0.45 | -2.28 | -0.22 | -1.30 | 0.05 | 0.82 | 0.36 | 1.24 | 0.25 | -2.20 |
|  | **T** | 2.08 | 0.01 | 1.28 | -2.07 | -1.19 | 1.65 | -0.34 | -0.71 | -2.83 | -0.52 | -0.34 | -1.38 | 0.17 | -0.26 | 0.57 | 1.40 | 0.41 | 0.80 | -1.01 | -2.59 |
|  | **V** | 1.52 | 0.03 | 1.60 | -2.89 | -1.43 | 1.99 | 0.57 | -1.07 | -2.89 | -1.23 | -0.03 | -1.14 | -0.19 | 0.24 | 0.69 | 1.87 | 1.41 | 0.19 | -0.19 | -2.64 |
|  | **W** | 2.33 | 1.24 | 1.03 | -1.01 | -0.70 | 0.85 | 0.70 | -0.27 | -1.97 | 0.15 | 0.31 | -0.82 | 0.08 | 0.99 | 0.39 | 3.23 | 0.27 | 0.56 | 0.73 | -0.92 |
|  | **Y** | 1.58 | 0.15 | 1.48 | -0.97 | -0.17 | 1.18 | 1.67 | 0.09 | -1.90 | -0.26 | 0.16 | -1.44 | -0.21 | -0.02 | 0.91 | 3.18 | 1.04 | 0.71 | -0.11 | -0.62 |
| **b)** | **p2m/p2p** | | | | **Mesophiles** | | | | | | | | | | | | | | | | |
|  |  | **A** | **C** | **D** | **E** | **F** | **G** | **H** | **I** | **K** | **L** | **M** | **N** | **P** | **Q** | **R** | **S** | **T** | **V** | **W** | **Y** |
| **Psychrophile** | **A** | -1.70 | 0.47 | -1.42 | 2.21 | 1.29 | -0.77 | 0.45 | 0.50 | 2.04 | 0.84 | 0.51 | 1.81 | 0.82 | 1.10 | -0.18 | -1.16 | -0.59 | -0.16 | 1.22 | 2.04 |
|  | **C** | -0.71 | -0.39 | -0.14 | 0.69 | 1.21 | -0.51 | -1.20 | 0.76 | 2.26 | 0.73 | 0.69 | 0.54 | 0.02 | 0.20 | -0.35 | -0.89 | -0.15 | 0.24 | 0.35 | 1.24 |
|  | **D** | -3.45 | -0.41 | -1.12 | 4.04 | 2.62 | -1.77 | 2.19 | 0.69 | 2.91 | 0.15 | 1.32 | 2.71 | 1.21 | 1.67 | -0.27 | -3.29 | -1.73 | -2.77 | -0.28 | 1.92 |
|  | **E** | -3.12 | -0.14 | -3.14 | 3.04 | 1.92 | -2.09 | 0.11 | -0.84 | 2.66 | 0.63 | 0.95 | 2.07 | 0.72 | 0.96 | -0.51 | -4.98 | -2.50 | -2.03 | 0.94 | 2.66 |
|  | **F** | -1.98 | 0.20 | -0.82 | 1.47 | -0.17 | -0.87 | -3.92 | 0.31 | 2.32 | 0.74 | -0.63 | 1.46 | -0.19 | 0.04 | -0.83 | -1.96 | -0.88 | -0.79 | -0.11 | 2.62 |
|  | **G** | -1.69 | -0.11 | -0.90 | 1.52 | 1.36 | -1.02 | 2.12 | 0.66 | 2.34 | 0.95 | 0.33 | 2.49 | 0.53 | 1.53 | 0.51 | -0.49 | -0.10 | -0.25 | 0.17 | 2.76 |
|  | **H** | -2.28 | 0.28 | -1.32 | 2.57 | 2.63 | -1.77 | -0.73 | 0.34 | 2.31 | 1.01 | 0.04 | 2.05 | 0.75 | 0.89 | -0.81 | -4.86 | -0.57 | -0.95 | 0.23 | 3.27 |
|  | **I** | -1.59 | 0.57 | -0.93 | 2.70 | 1.77 | -1.32 | -2.43 | 0.51 | 2.40 | 1.37 | -0.30 | 1.57 | 0.25 | 0.10 | -0.81 | -1.54 | -1.19 | -0.69 | 1.44 | 3.03 |
|  | **K** | -2.58 | -0.18 | -1.59 | 2.06 | 1.82 | -1.86 | 1.04 | -0.91 | 1.68 | -0.02 | 0.04 | 1.21 | 0.80 | 0.93 | -0.18 | -3.28 | -2.85 | -1.20 | 0.18 | 3.37 |
|  | **L** | -2.22 | -0.36 | -2.13 | 2.76 | 2.08 | -1.56 | -2.08 | 0.32 | 2.68 | 0.50 | -0.73 | 1.99 | -0.18 | 0.16 | -0.86 | -3.34 | -1.50 | -1.25 | 1.37 | 2.00 |
|  | **M** | -1.53 | 0.43 | -0.43 | 2.47 | 1.86 | -1.48 | -1.25 | 1.22 | 2.28 | 1.16 | -0.29 | 1.39 | 0.51 | 0.28 | -0.90 | -1.10 | -0.43 | -0.23 | 1.17 | 2.75 |
|  | **N** | -2.38 | -1.30 | -2.05 | 1.53 | 1.56 | -1.62 | 1.65 | 0.12 | 2.14 | 0.34 | 1.48 | 0.62 | 0.40 | 1.07 | -0.18 | -2.76 | -1.48 | -0.78 | -0.18 | 1.97 |
|  | **P** | -3.95 | -0.30 | -1.57 | 1.52 | 1.74 | -1.44 | 0.03 | 0.73 | 2.34 | 0.90 | 0.03 | 1.50 | 0.01 | -0.71 | -0.15 | -1.68 | -0.86 | -0.44 | 0.02 | 2.14 |
|  | **Q** | -2.91 | 1.17 | -2.86 | 2.95 | 2.03 | -2.19 | 0.57 | -0.22 | 2.28 | 0.81 | 1.03 | 1.81 | -0.44 | -0.02 | -0.49 | -4.65 | -2.60 | -1.57 | 0.23 | 3.07 |
|  | **R** | -2.24 | -0.55 | -1.05 | 1.23 | 1.34 | -1.70 | 0.37 | -0.20 | 1.65 | 0.40 | 0.26 | 1.02 | -0.25 | 0.50 | 0.25 | -2.37 | -1.16 | -0.57 | 0.88 | 2.23 |
|  | **S** | -2.63 | -0.19 | -2.40 | 3.47 | 2.13 | -1.64 | 0.14 | 0.46 | 2.68 | 0.62 | 0.80 | 2.33 | 0.83 | 1.04 | 0.07 | -1.29 | -1.12 | -1.04 | 0.74 | 3.09 |
|  | **T** | -2.65 | -0.43 | -2.80 | 4.19 | 1.86 | -1.57 | 0.70 | 1.16 | 2.81 | 0.78 | 0.54 | 1.56 | 0.48 | 0.98 | 0.03 | -3.00 | -0.40 | -0.74 | 1.07 | 3.09 |
|  | **V** | -1.81 | 0.17 | -2.03 | 3.46 | 1.23 | -1.02 | -1.03 | 0.86 | 2.67 | 0.65 | 0.09 | 1.76 | 0.17 | 0.44 | -0.59 | -1.34 | -1.01 | -0.03 | 0.84 | 2.08 |
|  | **W** | -0.87 | -0.37 | -0.89 | 0.94 | 0.78 | -0.22 | -0.44 | 0.13 | 1.55 | -0.20 | 0.02 | 0.29 | -0.58 | -1.08 | -0.28 | -3.42 | -0.33 | -0.21 | -1.38 | 1.01 |
|  | **Y** | -1.71 | -1.00 | -1.36 | 1.36 | -0.14 | -1.12 | -4.04 | 0.56 | 2.42 | 0.53 | 0.03 | 1.44 | -0.54 | -0.16 | -0.84 | -3.52 | -1.30 | -1.25 | 0.25 | 0.04 |
| The t-values calculated using LOS substitution scores of mutation frequencies normalized with substitution frequencies within the (a) mesophiles (m2m) and (b) psychrophiles (p2p) | | | | | | | | | | | | | | | | | | | | | |
