# Supplementary material for: Comparative proteome analysis of psychrophilic versus mesophilic bacterial species: Insights into the molecular basis of cold adaptation of proteins
Source: BMC Genomics. 2009 Jan 8;10:11. doi: 10.1186/1471-2164-10-11 (PMC2653534; doi:10.1186/1471-2164-10-11)
Supplement: Additional file 2 — T-test values for amino acid property groups' substitution preferences between proteins of Mesophilic and Psychrophilic proteomes. The t-values calculated using LOS substitution scores of mutation frequencies normalized with substitution frequencies within the mesophiles [file 1471-2164-10-11-S2.doc]

| **Additional file 2:** T-test values for amino acid property groups’ substitution preferences between proteins of Mesophilic and Psychrophilic proteomes. | | | | | | | | | | | | | |
| --- | --- | --- | --- | --- | --- | --- | --- | --- | --- | --- | --- | --- | --- |
| **Psychrophiles** | | | | | | | | | | | | | |
|  |  | **T** | **S** | **Al** | **Ar** | **Np** | **P** | **C** | **B** | **A** | **N** | **Hl** | **Hb** |
| **Mesophiles** | **Tiny (T)** | 1.964 | 1.967 | -0.434 | -1.942 | 1.561 | -1.127 | -1.954 | -2.813 | -0.824 | 1.556 | -2.344 | 1.292 |
|  | **Small (S)** | 3.590 | 2.166 | -0.806 | -2.076 | 1.232 | -1.654 | -2.372 | -2.800 | -0.886 | 3.359 | -3.510 | 0.838 |
|  | **Aliphatic (Al)** | 2.067 | 1.953 | -0.801 | -1.830 | -1.819 | -0.064 | -1.320 | -1.399 | -1.141 | 1.977 | -1.443 | -1.734 |
|  | **Aromatic(Ar)** | 2.258 | 1.394 | 0.028 | -0.273 | -0.035 | 0.173 | -0.803 | -0.519 | -0.346 | 2.522 | -1.369 | -0.178 |
|  | **Nonpolar (Np)** | 3.749 | 2.535 | -2.518 | -2.330 | 0.209 | -0.688 | -1.599 | -2.114 | -0.842 | 2.412 | -1.944 | -0.253 |
|  | **Polar (P)** | 4.635 | 3.827 | 0.391 | -3.987 | 1.085 | -1.781 | -3.378 | -4.020 | -1.645 | 2.357 | -3.827 | 1.153 |
|  | **Charged (C)** | 3.105 | 2.528 | 0.365 | -4.711 | 0.873 | -1.986 | -2.230 | -2.666 | -1.376 | 1.598 | -2.989 | 0.996 |
|  | **Basic (B)** | 2.712 | 1.766 | 0.258 | -3.029 | 0.560 | -1.473 | -1.940 | -1.537 | -0.654 | 1.434 | -2.475 | 0.510 |
|  | **Acidic (A)** | 3.336 | 3.244 | 0.489 | -1.698 | 1.228 | -2.290 | -2.382 | -2.223 | -0.842 | 1.392 | -3.177 | 1.588 |
|  | **Neutral (N)** | 3.427 | 3.694 | 0.109 | -2.764 | 1.746 | -0.877 | -1.976 | -3.088 | -0.717 | 1.130 | -2.412 | 0.878 |
|  | **H-philic (Hi)** | 3.505 | 3.091 | 0.590 | -2.508 | 1.199 | -2.046 | -2.855 | -3.737 | -1.329 | 1.968 | -2.906 | 1.415 |
|  | **H-phobic (Hb)** | 4.063 | 2.725 | -2.251 | -1.980 | -0.252 | -0.453 | -1.599 | -1.844 | -1.113 | 1.845 | -1.954 | -0.360 |
|  | **All** | 3.282 | 2.594 | -0.604 | -1.901 | 0.742 | -1.331 | -2.253 | -2.642 | -1.048 | 1.905 | -2.761 | 0.449 |
| The t-values calculated using LOS substitution scores of mutation frequencies normalized with substitution frequencies within the mesophiles | | | | | | | | | | | | | |
